# Associations between cohort derived dementia and COVID-19 serological diagnosis among older Black adults in rural South Africa

**DOI:** 10.3389/fpubh.2023.1304572

**Published:** 2024-01-05

**Authors:** Nigel Walsh Harriman, Darina T. Bassil, Meagan T. Farrell, Jacques Du Toit, F. Xavier Gómez-Olivé Casas, Stephen M. Tollman, Lisa F. Berkman

**Affiliations:** ^1^Harvard Center for Population and Development Studies, Harvard T.H. Chan School of Public Health, Cambridge, Massachusetts, MA, United States; ^2^MRC/Wits Rural Public Health and Health Transitions Research Unit (Agincourt), School of Public Health, Faculty of Health Sciences, University of the Witwatersrand, Johannesburg, Gauteng, South Africa

**Keywords:** COVID-19, older Black adults, South Africa, rural areas, Alzheimer’s disease and related dementias

## Abstract

**Objectives:**

This study investigates the association between cohort derived dementia and serologically confirmed SARS-CoV-2 infection, an underexplored phenomena in low-and middle-income countries. Examining this relationship in a rural South African community setting offers insights applicable to broader healthcare contexts.

**Methods:**

Data were collected from Black South Africans in the Mpumalanga province who participated in the Health and Aging in Africa: A Longitudinal Study of an INDEPTH Community in South Africa. Cohort derived dementia was developed using a predictive model for consensus-based dementia diagnosis. Multinomial logistic regression models estimated the association between predicted dementia probability in 2018 and SARS-CoV-2 infection risk in 2021, controlling for demographics, socioeconomic status, and comorbidities.

**Results:**

Fifty-two percent of the tested participants had serologically confirmed SARS-CoV-2 infections. In the fully adjusted model, cohort derived dementia was significantly associated with over twice the risk of serological diagnosis of COVID-19 (RRR = 2.12, *p* = 0.045).

**Conclusion:**

Complying with COVID-19 prevention recommendations may be difficult for individuals with impaired cognitive functioning due to their symptoms. Results can inform community-based public health initiatives to reduce COVID-19 transmission among South Africa’s rapidly aging population.

## Introduction

In Sub-Saharan Africa, the number of people aged over 60 years old is projected to more than double between 2015 and 2050 (1). With this unprecedented aging rate, it is estimated that nearly 3.48 million people across sub-Saharan Africa will have dementia by 2030, increasing further to 7.62 million people in 2050 (2). The year 2020 marked a significant turning point, as the burden of cognitive impairment and dementia became even more pronounced with the outbreak of the coronavirus disease (COVID-19) pandemic. Individuals with Alzheimer’s disease and related dementias (ADRD), already among society’s most vulnerable groups, have faced an elevated risk of SARS-CoV-2 infection (3, 4). This heightened risk mainly stemmed from difficulties in adopting preventative behaviors to reduce infection risk (5), and potential disruptions in care and services that may have disproportionately affected individuals with ADRD (6). Moreover, the presence of common comorbidities among older adults with ADRD may have also exacerbated the likelihood of mortality among those infected with SARS-CoV-2 (7). While extant research has linked ADRD with COVID-19 infection in upper-middle and high-income countries, such as China (8), the United Kingdom (9, 10), Belgium (11), and the United States (7), there remains a significant dearth of investigation into this relationship within low-and middle-income countries (LMICs).

Existing work on the association between ADRD and COVID-19 outcomes has overwhelmingly occurred in clinical settings (12). Examining this relationship in community settings can offer valuable insights applicable to health practitioners, policymakers, and researchers with findings that are generalizable to populations outside clinical settings. Such insights can extend beyond the scope of COVID-19 to other infectious diseases as well.

In the present study, we examined the relationship between ADRD and COVID-19 within a rural South African community setting, aiming to shed light on this unique region and contribute to a broader global understanding of the implications associated with ADRD and COVID-19.

## Methods

### Data source

Data were analyzed from Health and Aging in Africa: A Longitudinal Study of an INDEPTH Community in South Africa (HAALSI). HAALSI is a longitudinal population-based observational survey that aims to describe the physical and cognitive health of Black South Africans aged 40 and older in the Mpumalanga province. The study was conducted in the Agincourt sub-district in Mpumalanga Province, South Africa. The HAALSI cohort is nested within the Agincourt Health and Demographic Surveillance System (HDSS), which maintains a research platform that fully enumerates the population living across 31 villages (population over 116,000) in rural Mpumalanga Province (13). The Agincourt sub-district is characterized by the convergence of infectious and non-communicable diseases, a challenge exacerbated by the poor healthcare infrastructure. The region still faces difficulties in accessing basic amenities, including electricity and piped water. During apartheid, the Agincourt area was designated for the resettlement of Black South Africans, resulting in enforced racial segregation. While there have been some improvements in economic and educational opportunities since the end of apartheid in 1994, the area still grapples with low-income levels, high unemployment rates and an older population that had limited access to education in their early years.

A detailed description of the sampling methodology is described elsewhere (14). Participants were drawn from the existing sampling frame of the Agincourt Health and Socio-Demographic Surveillance System (Agincourt HDSS) site (13). Individuals were eligible for selection into the study if they were 40 years and older as of July 1, 2014, and permanently living in the study site during the year before the 2013 Agincourt census update (*n* = 6,281).

The first wave of data collection was conducted between November 2014 and November 2015. In-person interviews were administered using computer-assisted personal interviewing (CAPI) to a total of 5,059 respondents (85.9% response rate). Survey items were initially developed in English and then translated into the local language, xiTsonga. Wave 2 of the survey took place between October 2018 and November 2019; 94% of the living eligible participants from Wave 1 responded to this survey (*n* = 4,176). Data for Wave 3 of HAALSI were collected between July 2021 and March 2022; 94% of the living eligible participants from Wave 2 responded to this survey (*n* = 3,707).

### Variables

#### Cohort derived dementia

The exposure of interest in this study is an individual’s predicted probability of dementia at Wave 2. Following the approach used in the Aging Demographics and Memory Study of the Health and Retirement Study (HRS-ADAMS) (15), we developed a predictive model for dementia within the HAALSI-Dementia (HAALSI-HCAP) sub-cohort (*n* = 635) as a “gold standard” (16). We used logistic regression models to predict consensus-based dementia diagnosis (17). We then applied the model with the highest performance based on the maximum area under the Receiver Operating Characteristic Curve (ROC) to the parent HAALSI population to obtain a predicted dementia probability. This model (ROC = 0.79) included cognitive measures that are available in every HAALSI wave (immediate and delayed word recall, orientation score, activities of daily living, and self-rated memory) and those available from Wave 2 onward (verbal fluency, sum score of days of the week forward and backward, and instrumental activities of daily living).

#### COVID-19 serology result

The outcome of interest in this study, COVID-19 diagnosis at Wave 3, is a categorical variable with the following categories: COVID-19 Positive, Negative, Alive without serology test, and Deceased. During the Wave 3 HAALSI survey, nurses collected venous blood samples from consenting participants. Subsequently, two enzyme-linked immunosorbent assays (ELISA) were employed to assess the presence of antibodies to the SARS-CoV-2 spike trimer and nucleocapsid proteins (18). If either of these assays indicated the presence of SARS-CoV-2 related proteins, the participant was categorized as COVID-19 Positive; otherwise, they were classified as COVID-19 Negative. An individual was defined as “Deceased” if they (1) did not have a COVID-19 Serology Result, and (2) were confirmed as deceased before Wave 3, or (3) if there was census-linked date of death in between when they were contacted for the Wave 3 survey and when they were contacted for their COVID-19 serology test. All other respondents – those without a serology result and had not been confirmed to be deceased at the time of data collection – were categorized as “Alive without serology test.”

#### Covariates

Our analyses controlled for age, self-identified sex, employment status, educational attainment, household wealth, HIV status, depression, hypertension status, and diab0etes status. Unless noted, all covariates were assessed at Wave 2. Age was treated as a continuous variable and self-identified sex was binary (Male vs. Female). Regarding our socioeconomic variables, employment status was modeled as a binary variable (Not working/Retired vs. Employed/Home manager), educational attainment was measured at Wave 1 as an ordinal variable (no formal education, 1–7 years of education, 8–11 years, 12 or more years). Household wealth was measured using weighted scores derived from principal components analysis of household ownership of consumer durables (e.g., television, refrigerators, vehicles), livestock, and housing characteristics (e.g., sanitation facilities), with higher scores indicating greater wealth. The distribution of these weighted scores was then divided into quintiles (19). Depression was operationalized as a continuous variable using scores from the 20-item Center for Epidemiologic Studies Depression Scale (CES-D), with higher scores indicating increased symptoms of depression (20). An individual was defined as HIV positive if a capillary blood sample, or “dried blood spots” test indicated the presence of HIV antibodies at either Wave 1 or Wave 2. Participants were defined as diabetic if at either Wave 1 or Wave 2 they (1) self-reported they had been previously diagnosed with the condition or (2) had their random blood glucose measured with a Caresens^©^ N Monitor point-of-care machine with a reading equal to or over 11.1 mmol/L. Finally, participants were defined as hypertensive if at either Wave 1 or Wave 2 they (1) self-reported a previous diagnosis or (2) had a mean blood pressure reading of over 140/90 mmHg across three measurements at two-minute intervals.

### Analyses

We calculated the descriptive statistics (percentage, means, standard deviations, medians, and ranges) of our sample. Next, we assessed if the means of our continuous predictors varied by our dependent variable using Analyses of Variance (ANOVA) and the distribution of our categorical variables with Chi-Squared tests. Although the prevalence of missing data was low (5%), we imputed missing values for our covariates using multiple imputation with chained equations (MICE) to maintain the power of our analyses (21). We produced and performed analyses on 10 imputations of our dataset (22). Following this, we fit three multinomial logistic regression models to estimate the relative risk of testing positive for COVID-19 versus testing negative using relative risk ratios (RRR). Model 1 contained parameters for cohort derived dementia, age, and self-identified sex. Model 2 included all the parameters from Model 1, as well as employment status, educational attainment, and household wealth quintile. Finally, Model 3 contained all the variables from Model 2, and included variables for hypertension, diabetes, HIV status, and CES-D score.

## Results

### Sample description

A description of the sample is provided in Table 1. At the time of the COVID-19 serology test collection, between July 2021 and March 2022, 12% of the sample was confirmed to be deceased (*n* = 406), and 35% of the sample refused testing or could not be contacted. Among those with a test (*n* = 1,804), 52% were positive (*n* = 934). Predicted dementia probabilities were skewed to the right; the mean of the sample was 0.16 (SD = 0.2) and the median was 0.01 (range 0.01–0.89).

**Table 1:** Sample description (*n* = 3,372).

COVID-19 serology status	*N*	Percentage
Negative	870	25.8%
Positive	934	27.7%
Alive without serology test	1,162	34.5%
Deceased	406	12.0%

	*N*	Mean (SD)	Median (Range)	*p*-value^a^
Cohort derived dementia probability	3,372	0.157 (0.161)	0.095 (0.008–0.89)	<0.001
Age	3,372	67.14 (10.84)	66 (50–115)	<0.001
CES-D score	3,363	14.56 (9.35)	13 (0–51)	0.003

	*N*	Percentage	*p*-value^a^
Self-identified sex			
Male	1,485	44.0%	<0.001
Female	1,887	56.0%	
Employment status			
Not working or retired	2,913	86.4%	<0.001
Employed or home manager	452	13.40%	
Missing	7	0.2%	
Education attainment			
No formal education	1,574	46.7%	0.002
Some primary (1–7 years)	1,226	36.4%	
Some secondary (8–11 years)	330	9.8%	
Secondary or more (12+ years)	234	6.9%	
Missing	8	0.2%	
Household wealth quintile			
Q1 (Poorest)	679	20.1%	0.07
Q2	658	19.5%	
Q3	638	18.9%	
Q4	679	20.1%	
Q5 (Wealthiest)	718	21.3%	
HIV status			
Negative	2,520	74.7%	0.02
Positive	722	21.4%	
Missing	130	3.9%	
Hypertension status			
Not hypertensive	852	25.3%	0.01
Hypertensive	2,499	74.1%	
Missing	21	0.6%	
Diabetes status			
Not diabetic	2,668	79.1%	<0.001
Diabetic	600	17.8%	
Missing	104	3.1%	

^a^*p*-values correspond to Chi-Squared tests for categorical variables and ANOVA tests for continuous variables.

The mean age was 67.1 (SD 10.8) and there were more women (56%) than men (44%) in the sample. The majority of participants were not working or retired (87%) and approximately half of the sample had no formal education (47%). Regarding comorbidities, 22% of the sample had been diagnosed as HIV positive, 18% had been diagnosed with diabetes, and 75% of the sample had been diagnosed with hypertension. Finally, the mean CES-D score for the sample was 14.6 (SD 9.4), and 35% of the sample scored higher than the clinical cutoff of 16. With the exception of household wealth quintile (*p* = 0.07), all variables were significantly associated with one’s predicted probability of dementia in univariate analyses.

### Multinominal logistic regression models

Results from our multinomial logistic regression models describing the association between cohort derived dementia and COVID-19 serological diagnosis can be found in Table 2. In model 1, which adjusts for age and self-identified sex, we observe that predicted dementia is associated with a two-fold increase in the relative risk of testing positive for COVID-19, versus testing negative, holding all other covariates constant (RRR = 2.05, *p* = 0.047). With the addition of our proxies of socioeconomic status in model 2, the association between cohort derived dementia and positive serology test remains significant. In this model, predicted dementia is associated with a 111% increase in the relative risk of testing positive for COVID-19 (RRR = 2.11, *p* = 0.046). Model 3 includes all the parameters from model 2, but also adjusts for the following lifetime comorbidities measured at Wave 2: HIV, Diabetes, Hypertension, and CES-D score. In this model, predicted dementia was associated with 2.12 times the relative risk of testing positive for COVID-19, compared to testing negative (RRR = 2.12, *p* = 0.045).

**Table 2:** Multinomial logistic regression coefficients comparing positive versus negative COVID-19 serology result (*n* = 3,372).

Variables	Model 1^a^			Model 2^b^			Model 3^c^		
	RRR	SE	*p*-value	RRR	SE	*p*-value	RRR	SE	*p*-value
Cohort derived dementia	**2.050**	**0.740**	**0.047**	**2.109**	**0.788**	**0.046**	**2.119**	**0.795**	**0.045**
Age	**0.980**	**0.005**	**<0.001**	**0.982**	**0.005**	**0.001**	**0.980**	**0.006**	**<0.001**
Male	Ref			Ref			Ref		
Female	**1.326**	**0.130**	**0.004**	**1.348**	**0.134**	**0.003**	**1.354**	**0.135**	**0.002**
Not working/Retired				Ref			Ref		
Employed/Home manager				**1.507**	**0.241**	**0.010**	**1.504**	**0.240**	**0.011**
No formal education				Ref			Ref		
Some primary (1–7 years)				0.804	0.090	0.052	**0.796**	**0.090**	**0.043**
Some secondary (8–11 years)				0.982	0.175	0.920	0.981	0.175	0.913
Secondary or more (12+ years)				0.723	0.165	0.156	0.709	0.163	0.134
Household wealth Q1 (Poorest)				Ref			Ref		
Q2				1.145	0.176	0.379	1.143	0.175	0.384
Q3				1.214	0.188	0.212	1.218	0.189	0.203
Q4				1.297	0.196	0.084	1.288	0.195	0.094
Q5 (Wealthiest)				**1.490**	**0.237**	**0.012**	**1.466**	**0.234**	**0.017**
CES-D score							0.998	0.005	0.656
HIV negative							Ref		
HIV positive							0.836	0.102	0.141
No hypertension							Ref		
Hypertensive							0.952	0.109	0.670
No diabetes							Ref		
Diabetic							1.077	0.135	0.554

^a^Cohort derived dementia + Age & Self-identified sex.

^b^Model 1 + SES.

^c^Model 2 + Comorbidities.

Bold indicates significance at the 0.05 alpha level.

In our final model, we observed that age was negatively associated with testing positive for COVID-19 (RRR = 0.98, *p* < 0.001) and women had 1.35 times the relative risk of testing positive for COVID-19 than men (RRR = 1.35, *p* = 0.002). Compared to those who were not working or retired, individuals who were employed or home managers had 1.5 times the risk of testing positive for COVID-19 relative to testing negative (RRR = 1.5, *p* = 0.011). Compared to individuals with no formal education, those with some primary education (1–7 years) had a 20% decreased risk of testing positive for COVID-19, relative to testing negative (RRR = 0.8, *p* = 0.043). Additionally, individuals who were in the highest quintile of household wealth had 1.47 times the relative risk of testing positive for COVID-19, compared to those in the poorest quintile (RRR = 1.47, *p* = 0.017). None of the lifetime comorbidities were significantly associated with the risk of testing positive for COVID-19 relative to testing negative.

### Sensitivity analyses

We performed several sensitivity analyses, including replicating the models with complete case analysis and adjusting for comorbidity status at Wave 2 only. Our reported results remained largely consistent throughout the sensitivity analyses. The only divergence occurred within our complete case analyses, where predicted dementia was marginally associated with COVID-19 diagnosis in our final model (*p* = 0.059). Full results from these sensitivity analyses can be found in the Supplementary material.

## Discussion

Our aim was to present a comprehensive analysis examining the association between the cohort derived dementia in 2018–19 and subsequent COVID-19 serological diagnosis in 2021–22. To our knowledge, this is the first study within South Africa to examine the potential association between dementia and serologically confirmed SARS-CoV-2 infection. Our findings reveal a significant association between cohort derived dementia and serological diagnosis of COVID-19.

Our results align with the growing body of literature on the relationship between ADRD and risk for SARS-CoV-2 infection – all of which has been conducted outside of South Africa. In a landmark retrospective study of 61.9 million electronic health records in the United States, Wang et al. reported that patients with dementia had twice the risk of SARS-CoV-2 infection compared to those without the disease (7). Consistent with our study, the association between ADRD and the risk of SARS-CoV-2 infection was independent of an individual’s age. Our results regarding the magnitude of risk of SARS-CoV-19 infection among those who have ADRD are also in accordance with existing literature. In addition to the Wang et al. analysis, cohort studies conducted in the United Kingdom, using the UK Biobank dataset, indicated all-cause dementia was associated with nearly threefold odds of SARS-CoV-2 infection (9). Other extant work in care facilities and community living centers in Sweden and the United States has underscored the significance of ADRD as an important risk factor for both SARS-CoV-2 infection and COVID-19 mortality (23, 24).

There are several explanations offered in the existing literature as to why individuals who have impaired cognitive function and/or ADRD are at an increased risk for SARS-CoV-2 infection. Specifically, these individuals can face difficulties in adhering to recommended preventive measures against the spread of COVID-19. Existing work has suggested that individuals with impaired cognitive function and/or ADRD may forget to engage in handwashing and mask-wearing, and those with more progressive forms of the disease may have difficulties with complying with social distancing recommendations due to wandering (25, 26). As evidenced by the high rates of SARS-CoV-2 infection among patients with ADRD in care facilities and living centers (23, 24, 27, 28), physical distancing compliance is difficult for those who require assistance from healthcare providers in their activities of daily living, especially if personal protective equipment (PPE) is in short supply for providers. Moreover, in rural South Africa, multi-generational housing is common, and care for chronically sick individuals in the household is typically delivered by adults in the household (29, 30). However, informal caretakers like these are less likely to possess the knowledge of healthcare workers regarding safeguarding measures and practices to ensure the well-being of individuals with ADRD against SARS-CoV-2 infection. This is especially important to consider given that social distancing is no longer required, as individuals with ADRD may remain vulnerable to exposure via their informal caregivers. As such, providers of care should be well-informed that they are working with an at-risk population.

Despite the emerging consensus in the literature that ADRD place individuals at heightened risk for SARS-CoV-2 infection, our work adds a novel contribution to the field in two ways. First, in contrast to the aforementioned studies utilizing clinical data, our study sample was obtained from a population-based survey in rural South Africa. Second, to our knowledge, our study represents a pioneering effort to investigate the association between ADRD and risk for SARS-Cov-2 infection in South Africa. While the susceptibility of aging populations, especially those with ADRD, to SARS-CoV-2 infection has received attention in South Africa (28, 31), existing quantitative work has predominantly focused on its association with HIV/AIDS, hypertension, and diabetes (32, 33). Understanding how ADRD is related to SARS-CoV-2 infection beyond clinical settings is vital for shaping public health policies and preventive strategies against the spread of COVID-19, and infectious diseases in general. The fact that our study takes place outside the clinical setting adds unique relevance to our findings for policymakers and practitioners, considering that the majority of COVID-19 cases are now identified outside of hospital settings.

Our analysis has several limitations. Primarily, we cannot make causal claims about the associations presented. Although our analysis established a robust association between cohort derived dementia and COVID-19 diagnosis, we are unable to assess its relation to infection severity. Although prior research has linked ADRD to COVID-19 morbidity and mortality, our data were limited in their ability to assess this in our sample (24, 34–36). Moreover, prior research in Agincourt during a similar period (August 2021) indicated high rates of asymptomatic cases (approximately 85% of infected individuals) (27). While the determinants of SARS-CoV-2 infection remain an important metric for disease control, future research should aim to assess whether ADRD are associated with COVID-19 morbidity and mortality. Data collection for this study spanned from July 2021 and May 2022, with 97% of the data collected between August 2021 and December 2021. As such, we have limited information on the experiences of those with an increased risk of dementia outside of the end of the Delta (May 2021–November 2021), and the beginning of the Omicron (November 2021–December 2022) waves in South Africa (37, 38). Finally, it is important to acknowledge that our sample is broadly representative of older, Black populations in low-income rural regions of South Africa. While providing valuable insights, this sample does not offer a comprehensive representation of the entire spectrum of the South African population.

Limitations notwithstanding, our work has important implications for future research, clinical practice, and policy. Existing research on the best practices for improving the quality of care for people with ADRD living at home has recommended the implementation of educational interventions for their caregivers (39). Recent work during the COVID-19 pandemic has recommended community-based outreach to deliver educational materials, such as creating hotlines for caregivers to access when help is needed; however, this may need to be adapted to the rural South African context (26, 40). For example, leveraging commonly utilized frameworks, including door-to-door outreach by community health workers, may be an avenue for delivering educational initiatives. Community outreach through this approach has been effective in reducing the burden of other non-communicable diseases in South Africa and thus is a compelling option to apply to the ADRD population (41, 42). Although great progress has been made in reducing the COVID-19-related burden of disease, it remains vital to consider targeted public health interventions for those with ADRD, because of their vulnerability for morbidity, mortality, and breakthrough infection (34, 36, 43).

To our knowledge, this work is the first to establish an association between ADRD and SARS-CoV-2 infection in South Africa. Our finding that cohort derived dementia is associated with an increased risk of SARS-CoV-2 infection, independent of age, aligns well with existing research on the topic. These results are vital to the development of public health initiatives, practices, and policies designed to reduce COVID-19 transmission, morbidity, and mortality among South Africa’s rapidly aging population.

## Data availability statement

The datasets presented in this study can be found in online repositories. The names of the repository/repositories and accession number(s) can be found at: https://haalsi.org/data.

## Ethics statement

The studies involving humans were approved by Harvard T.H. Chan School of Public Health. The studies were conducted in accordance with the local legislation and institutional requirements. The participants provided their written informed consent to participate in this study.

## Author contributions

NH: Formal analysis, Methodology, Writing – original draft. DB: Methodology, Supervision, Writing – original draft, Writing – review & editing. MF: Methodology, Supervision, Writing – review & editing. JT: Methodology, Supervision, Writing – review & editing. FG-O: Data curation, Methodology, Supervision, Writing – review & editing. ST: Conceptualization, Investigation, Supervision, Writing – review & editing. LB: Conceptualization, Investigation, Writing – review & editing.
