# Supplementary material for: Associations between cohort derived dementia and COVID-19 serological diagnosis among older Black adults in rural South Africa
Source: Front Public Health. 2024 Jan 5;11:1304572. doi: 10.3389/fpubh.2023.1304572 (PMC10796535; doi:10.3389/fpubh.2023.1304572)
Supplement: Supplementary file 1 [file Table_1.docx]

**Multinomial Logistic Regression Coefficients Comparing Positive versus Negative COVID-19 Serology Result – Complete Case Analysis and Wave 2 Comorbidity**

|  | **Model 1^a^**  **n=3,372** | | | **Model 2^b^**  **n=3,357** | | | **Model 3^c^**  **n=3,201** | | | **Model 4^d^**  **n=2,632** | | |
| --- | --- | --- | --- | --- | --- | --- | --- | --- | --- | --- | --- | --- |
| **VARIABLES** | **RRR** | **SE** | ***p*-value** | **RRR** | **SE** | ***p*-value** | **RRR** | **SE** | ***p*-value** | **RRR** | **SE** | ***p*-value** |
| Cohort Derived Dementia | **2.050** | **0.740** | **0.047** | **2.167** | **0.812** | **0.039** | 2.058 | 0.788 | 0.059 | 2.043 | 0.842 | 0.083 |
| Age | **0.980** | **0.005** | **<0.001** | **0.982** | **0.005** | **0.001** | **0.981** | **0.006** | **0.001** | **0.980** | **0.006** | **0.001** |
| Male | *ref* | | | *ref* | | | *ref* | | | *ref* | | |
| Female | **1.326** | **0.130** | **0.004** | **1.354** | **0.135** | **0.002** | **1.367** | **0.140** | **0.002** | **1.404** | **0.154** | **0.002** |
| Not working / Retired |  |  |  | *ref* | | | *ref* | | | *ref* | | |
| Employed / Home manager |  |  |  | **1.510** | **0.241** | **0.010** | **1.518** | **0.252** | **0.012** | 1.391 | 0.245 | 0.062 |
| No formal education |  |  |  | *ref* | | | *ref* | | | *ref* | | |
| Some primary (1-7 years) |  |  |  | 0.812 | 0.091 | 0.064 | 0.819 | 0.094 | 0.081 | 0.848 | 0.104 | 0.178 |
| Some secondary (8-11 years) |  |  |  | 0.997 | 0.178 | 0.985 | 1.092 | 0.200 | 0.631 | 1.067 | 0.208 | 0.739 |
| Secondary or more (12+ years) |  |  |  | 0.726 | 0.166 | 0.162 | 0.646 | 0.153 | 0.065 | 0.632 | 0.161 | 0.072 |
| Household Wealth Q1 (Poorest) |  |  |  | *ref* | | | *ref* | | | *ref* | | |
| Q2 |  |  |  | 1.125 | 0.173 | 0.443 | 1.115 | 0.175 | 0.487 | 1.181 | 0.199 | 0.325 |
| Q3 |  |  |  | 1.207 | 0.188 | 0.226 | 1.231 | 0.196 | 0.192 | 1.335 | 0.231 | 0.095 |
| Q4 |  |  |  | 1.290 | 0.195 | 0.092 | 1.294 | 0.200 | 0.094 | 1.354 | 0.225 | 0.069 |
| Q5 (Wealthiest) |  |  |  | **1.489** | **0.237** | **0.012** | **1.476** | **0.241** | **0.017** | **1.525** | **0.267** | **0.016** |
| CES-D Score |  |  |  |  |  |  | 0.998 | 0.005 | 0.753 | 1.001 | 0.006 | 0.854 |
| HIV Negative |  |  |  |  |  |  | *ref* | | | *ref* | | |
| HIV Positive |  |  |  |  |  |  | 0.855 | 0.104 | 0.196 | 0.825 | 0.108 | 0.140 |
| No Hypertension |  |  |  |  |  |  | *ref* | | | *ref* | | |
| Hypertensive |  |  |  |  |  |  | 0.968 | 0.113 | 0.778 | 0.974 | 0.113 | 0.824 |
| No Diabetes |  |  |  |  |  |  | *ref* | | | *ref* | | |
| Diabetic |  |  |  |  |  |  | 1.088 | 0.138 | 0.504 | 1.058 | 0.146 | 0.684 |

**a – Cohort Derived Dementia + Age & Self-Identified Sex**

**b – Model 1 + SES**

**c – Model 2 + Lifetime Comorbidities**

**d – Model 2 + Wave 2 Comorbidities**
