# Supplementary material for: Associations between cohort derived dementia and COVID-19 serological diagnosis among older Black adults in rural South Africa
Source: Front Public Health. 2024 Jan 5;11:1304572. doi: 10.3389/fpubh.2023.1304572 (PMC10796535; doi:10.3389/fpubh.2023.1304572)
Supplement: Supplementary file 2 [file Table_2.docx]

**Multinomial Logistic Regression Coefficients Comparing Positive versus Negative COVID-19 Serology Result – Imputed Analysis and Wave 2 Comorbidity**

|  | **Model 1^a^**  **n=3,372** | | | **Model 2^b^**  **n=3,372** | | | **Model 3^c^**  **n=3,372** | | | **Model 4^d^**  **n=3,372** | | |
| --- | --- | --- | --- | --- | --- | --- | --- | --- | --- | --- | --- | --- |
| **VARIABLES** | **RRR** | **SE** | ***p*-value** | **RRR** | **SE** | ***p*-value** | **RRR** | **SE** | ***p*-value** | **RRR** | **SE** | ***p*-value** |
| Cohort Derived Dementia | **2.050** | **0.740** | **0.047** | **2.109** | **0.788** | **0.046** | **2.119** | **0.795** | **0.045** | **2.133** | **0.800** | **0.044** |
| Age | **0.980** | **0.005** | **<0.001** | **0.982** | **0.005** | **0.001** | **0.980** | **0.006** | **<0.001** | **0.980** | **0.006** | **<0.001** |
| Male | *ref* | | | *ref* | | | *ref* | | |  | | |
| Female | **1.326** | **0.130** | **0.004** | **1.348** | **0.134** | **0.003** | **1.354** | **0.135** | **0.002** | **1.353** | **0.136** | **0.003** |
| Not working / Retired |  |  |  | *ref* | | | *ref* | | |  | | |
| Employed / Home manager |  |  |  | **1.507** | **0.241** | **0.010** | **1.504** | **0.240** | **0.011** | **1.503** | **0.240** | **0.011** |
| No formal education |  |  |  | *ref* | | | *ref* | | |  | | |
| Some primary (1-7 years) |  |  |  | 0.804 | 0.090 | 0.052 | **0.796** | **0.090** | **0.043** | **0.800** | **0.090** | **0.048** |
| Some secondary (8-11 years) |  |  |  | 0.982 | 0.175 | 0.920 | 0.981 | 0.175 | 0.913 | 0.983 | 0.175 | 0.923 |
| Secondary or more (12+ years) |  |  |  | 0.723 | 0.165 | 0.156 | 0.709 | 0.163 | 0.134 | 0.712 | 0.163 | 0.138 |
| Household Wealth Q1 (Poorest) |  |  |  | *ref* | | | *ref* | | |  | | |
| Q2 |  |  |  | 1.145 | 0.176 | 0.379 | 1.143 | 0.175 | 0.384 | 1.140 | 0.175 | 0.392 |
| Q3 |  |  |  | 1.214 | 0.188 | 0.212 | 1.218 | 0.189 | 0.203 | 1.219 | 0.189 | 0.203 |
| Q4 |  |  |  | 1.297 | 0.196 | 0.084 | 1.288 | 0.195 | 0.094 | 1.288 | 0.195 | 0.094 |
| Q5 (Wealthiest) |  |  |  | **1.490** | **0.237** | **0.012** | **1.466** | **0.234** | **0.017** | **1.471** | **0.235** | **0.016** |
| CES-D Score |  |  |  |  |  |  | 0.998 | 0.005 | 0.656 | 0.998 | 0.005 | 0.675 |
| HIV Negative |  |  |  |  |  |  | *ref* | | |  | | |
| HIV Positive |  |  |  |  |  |  | 0.836 | 0.102 | 0.141 | 0.839 | 0.100 | 0.142 |
| No Hypertension |  |  |  |  |  |  | *ref* | | |  | | |
| Hypertensive |  |  |  |  |  |  | 0.952 | 0.109 | 0.670 | 0.986 | 0.110 | 0.898 |
| No Diabetes |  |  |  |  |  |  | *ref* | | |  | | |
| Diabetic |  |  |  |  |  |  | 1.077 | 0.135 | 0.554 | 1.013 | 0.137 | 0.922 |

**a – Cohort Derived Dementia + Age & Self-Identified Sex**

**b – Model 1 + SES**

**c – Model 2 + Lifetime Comorbidities**

**d – Model 2 + Wave 2 Comorbidities**
